# Intake of *Calanus finmarchicus* oil for 12 weeks improves omega-3 index in healthy older subjects engaging in an exercise programme

**DOI:** 10.1017/S0007114520002809

**Published:** 2021-02-28

**Authors:** Paulina Wasserfurth, Josefine Nebl, Tim Konstantin Boßlau, Karsten Krüger, Andreas Hahn, Jan Philipp Schuchardt

**Affiliations:** 1 Faculty of Natural Sciences, Institute of Food Science and Human Nutrition, Leibniz University Hannover, 30167 Hannover, Germany; 2 Department of Exercise Physiology and Sports Therapy, Institute of Sports Science, Justus-Liebig-University Giessen, 35394 Giessen, Germany

**Keywords:** PUFA, Marine oil, Wax esters, EPA, DHA

## Abstract

The *n*-3 PUFA, EPA and DHA, play an important role in human health. As the intake of EPA and DHA from the diet is often inadequate, supplementation of those fatty acids is recommended. A novel source of *n*-3 PUFA is *Calanus finmarchicus* oil (CO) which contains fatty acids mainly bound in wax esters. To date, no data are available on the effects of long-term intake of this marine oil on *n*-3 PUFA blood levels. Therefore, the aim of this study was to evaluate the effect of CO on the *n*-3 PUFA blood levels using the omega-3 index (O3I). The data originate from a larger randomised controlled trial. For this analysis, samples from seventy-two participants (59·2 (sd 6·2) years, BMI 27·7 (sd 5·28) kg/m^2^) were analysed. Of those, thirty-six performed 2×/week exercise and received 2 g of CO, which provided 124 mg stearidonic acid (SDA), 109 mg EPA and 87 mg DHA daily (EXCO group), while the other group performed exercise only (EX group) and served as a control for this analysis. The O3I increased from 6·07 (sd 1·29) % at baseline to 7·37 (sd 1·10) % after 12 weeks within the EXCO group (*P* < 0·001), while there were no significant changes in the EX group (6·01 (sd 1·26)–6·15 (sd 1·32) %, *P* = 0·238). These data provide first evidence that wax ester-bound *n*-3 PUFA from CO can significantly increase the O3I despite relatively low EPA + DHA amounts. Further, the effects of exercise could be excluded.

*n*-3 PUFA are highly associated with human health, playing an important role in cardiovascular health, brain development and cognitive function as well as inflammation and inflammatory diseases^(1,2)^.

Of all *n*-3 PUFA, most of those health benefits are attributed to the 20 : 5*n*-3 fatty acid EPA and the 22 : 6*n*-3 fatty acid DHA. Regarding their importance in human health and an unfavourable shift in the dietary fatty acid intake in favour of *n*-6 fatty acids, daily intake of at least 0·5 g of EPA and DHA is recommended by the International Society for the Study of Fatty Acids and Lipids (ISSFAL)^(3)^. The primary dietary source for EPA and DHA is cold-water fish such as tuna, salmon, herring or mackerel; however, intake of these types of fish is often low^(4)^. In Germany, this is also reflected by low median dietary intakes of 65–78 mg EPA and 107–135 mg DHA^(5)^. Therefore, supplementation of *n*-3 PUFA may be beneficial. Good *n*-3 PUFA sources that are commonly used for supplementation are marine oils, with fish oil being the most frequently used^(6)^. A novel *n*-3 PUFA source is the marine oil obtained from *Calanus finmarchicus*, a copepod found in the northern Atlantic sea. Unlike fish oil, where *n*-3 PUFA are bound in the form of TAG, >80 % of the fatty acids in *Calanus finmarchicus* oil (CO) are bound in the form of wax esters^(7)^. In any case, when compared with refined fish oil which commonly contains 300 mg EPA + DHA per g^(8)^, the amount of EPA and DHA in CO is relatively low (about 100 mg EPA + DHA per g). A single-dose study has already shown that EPA and DHA from CO are equally bioavailable as from fish oil^(9)^. However, the long-term intake of *n*-3 PUFA from CO has not yet been studied. The omega-3 index (O3I) (relative content of EPA + DHA in erythrocytes) has been shown to be a good and reliable indicator in evaluating the fatty acid supply over a longer period of time^(10,11)^.

In addition to being an indicator for the long-term fatty acid supply, an O3I of >8 % was also linked to a lower risk for cardiovascular events^(10,12)^. This is of particular interest for all age groups engaging in exercise, as exercise increases the demands of the cardiovascular system. Although it was already demonstrated that athletes show an insufficient supply with *n*-3 PUFA^(13,14)^, no data about the effects of exercise on the O3I have been described to date.

Hence, the objective of the present study was to investigate the effect of CO providing physiological EPA + DHA doses on the O3I in a study collective of healthy elderly subjects who participated in an exercise programme. To account for potential effects of the exercise programme on the O3I, the group performing exercise only served as a control.

## Materials and methods

### Study design and participants

The present work is based on a single-centre, randomised controlled trial in parallel-group design which was conducted at the Institute of Food Science and Human Nutrition, Leibniz University Hannover, Germany. In brief, the study consisted of a screening and 12-week intervention phase with two examination days; one at the beginning (*t*_0_) and one at the end of the 12-week intervention (*t*_12_).

Participants for this study were recruited via advertisements in local newspapers and public notice boards from the general population in Hannover, Germany, between August 2018 and March 2019. The trial ended when the required sample size was achieved. The main inclusion criteria for participation were age ≥50 and ≤70 years, no exercise training aside the daily activities for at least 2 years, a stable body weight (±5 kg) for at least 6 months, being able to physically perform the exercise intervention (exercise capacity) and following an omnivorous diet. Exclusion criteria were defined as: suspicion and diagnosis of CVD (angina pectoris, myocardial infarction, stroke, peripheral arterial occlusive disease, heart failure and cardiac arrhythmia), type 1 and 2 diabetes, renal insufficiency and liver diseases, blood coagulation disorders, chronic gastrointestinal disorders (e.g. ulcers, Crohn’s disease, pancreatic insufficiency, immunological diseases (e.g. autoimmune diseases)), intake of immunosuppressive drugs or laxatives, intake of supplements containing *n*-3 PUFA, alcohol, drug and/or medicine dependency, pregnancy or lactation, retraction of the consent by the subject, concurrent participation in another clinical study and participation in a study in the last 30 d. Inclusion and exclusion criteria were assessed using a structured screening questionnaire. Exercise capacity was determined during a resting and exercise electrocardiogram, implemented by trained professionals and a physician. This study was conducted according to the guidelines in the Declaration of Helsinki, and all procedures involving human subjects were approved by the Ethics Commission of the Medical Chamber of Lower Saxony (Hannover, Germany) (Bo/07/2018, URL: https://www.drks.de/drks_web/setLocale_EN.do). This study is registered in the German Clinical Trial Register (DRKS00014322).

The participants were randomly assigned by an independent researcher using stratified randomisation according to the covariates (in descending order: sex, BMI and age) to one of four study groups: (1) control group (CON), (2) exercise only group (EX), (3) exercise and dietary counseling group (EXDC) and (4) exercise and CO supplementation group (EXCO). However, the present work focuses on the EX and EXCO groups only.

Both groups were instructed to perform exercise training twice a week and maintain their habitual diet. The exercise training was performed in fitness centres and consisted of a warm up followed by two passes of a strength-endurance circuit. The strength training consisted of six machine supported exercises that included all major muscle groups and were performed for 1 min each. During the initial training session, a maximum force test with three tries was performed. The best of the three tries was scored and used to set the machines to 60 % of the participants’ maximum force for the first 2 weeks of training. For the subsequent 6 weeks, the load was increased by 10 % and again by 5 % for the last 4 weeks. The endurance exercise consisted of a 4-min bout performed on bicycle ergometers and cross-trainers at a perceived exertion that equaled a value of 15 on the Borg-Scale. In between each exercise, the participants had 30 s of rest. Including the warm-up and rest periods, the training session could be completed in approximately 1 hour. Compliance of the participants was assessed via a training log and a questionnaire at the end of the study.

In addition to the exercise training, participants from the EXCO group received capsules providing 2·0 g of oil from *Calanus finmarchicus* (Calanus AS) and were instructed to take them daily. The lipid profile of the capsules is shown in Table 1. More than 80 % of the fatty acids are bound as wax esters. The tolerability of the CO capsules was checked using questionnaires.

**Table 1.** Fatty acid composition of the *Calanus finmarchicus* oil (CO) used in the study

Fatty acid*	Common name	mg/100 g CO	mg/2 g CO
14 : 0	Myristic acid	6232	125
15 : 0	Pentadeclic acid	323	6·5
16 : 0	Palmitic acid	5243	105
16 : 3		325	7
18 : 0	Stearic acid	399	8
18 : 1*n*-9	Oleic acid	1783	36
18 : 2*n*-6	Linoleic acid	552	11
18 : 3*n*-3	*α*-Linolenic acid	1149	23
18 : 3*n*-6	*β*-Linolenic acid	140	3
18 : 4*n*-3	Stearidonic acid	6186	124
20 : 1*n*-9	Gondoic acid	2148	43
20 : 4*n*-6	Arachidonic acid	169	3
20 : 5*n*-3	EPA	5439	109
22 : 1*n*-11	Cetoleic acid	3495	70
22 : 5*n*-3	Docosapentaenoic acid	254	8
22 : 6*n*-3	DHA	4342	87
24 : 1*n*-9		414	8

* >80 % of fatty acids are present as wax esters.

Compliance of participants and adherence to the instructions were monitored via fortnightly phone calls. Additionally, participants from the EXCO were instructed to return leftover capsules, which were then counted by study personal after delivery. Participants had to consume at least 90 % of the capsules to be considered as compliant.

### Dietary intake from background diet

Dietary intake of the participants was monitored via 3-d dietary food logs at the beginning, after 6 weeks and at the end of the intervention. The records were checked by nutritionists for completeness, readability and plausibility. If necessary, ambiguities were clarified with the participants. Energy and nutrient intake were estimated using the software PRODI6.4® (Nutri-Science GmbH).

### Blood sampling and analysis

Blood samples were drawn from the participants after an overnight fast (≥10 h) between 06.00 and 10.00 hours by venepuncture of an arm vein using EDTA tubes (Sarstedt AG & Co. KG). TAG, LDL-cholesterol and HDL-cholesterol were analysed by a photometric method (Beckman Coulter GmbH) by an accredited and certified laboratory (Laborärztliche Arbeitsgemeinschaft für Diagnostik und Rationalisierung e.V.). Total cholesterol and LDL:HDL ratio were calculated from LDL and HDL values.

For analysis of O3I, EDTA tubes were centrifuged for 10 min at 3000 rpm, buffy coat was removed and erythrocytes frozen at –80°C till analysis. The analysis was performed by the Omegametrix laboratory (Martinsried) according to the HS-*n*-3 Index methodology®^(10,12)^ by GC. Accordingly, methyl esters of fatty acids were generated from erythrocytes by acid transesterification, and analysis performed using a GC2010 gas chromatograph (Shimadzu) equipped with a SP2560, 100 m column (Supelco). Hydrogen was used as the carrier gas. To identify fatty acids, a standard mixture characteristic for erythrocytes was used. The results obtained are given as percentage of total identified fatty acids after response factor correction. The O3I represents the sum of EPA + DHA in relation to total fatty acid content in red blood cell membranes. Quality was assured according to DIN ISO 15189.

### Statistical analyses

Data for this analysis were derived from a larger interventional trial, which served as an explorative study. Based on an *α* of 0·05 and 0·80 *β*, assuming an effect size of more than 0·8, a sample size of *n* 25 was needed to detect between-group differences. Estimating a dropout rate of 15 % at least thirty participants per intervention group were recruited. For this analysis, a power calculation was performed using an online calculator^(15)^. As CO was demonstrated to be equally bioavailable as ethyl esters^(9)^, the calculation was based on a study by Köhler *et al.*^(16)^. In the study by Köhler *et al.*^(16)^, participants received 250 mg ethyl-ester bound EPA + DHA in the form of enriched sausages which resulted in an O3I increase from 4·18 (sd 0·54) to 5·72 (sd 0·66) % after 8 weeks. Therefore, we assumed that a change of the O3I of at least 1 % could be achieved after 12 weeks of CO supplementation providing about 200 mg EPA + DHA. Using the online calculator with alpha set to 0·05 and *n* 50 total participants, assuming a mean difference of 1·0 and a 0·7 % standard deviation (using 5·72 (sd 0·66) % from Köhler *et al.*^(16)^ as the upper level for the standard deviation) a 99 % probability to detect a difference if the true difference between groups is 1 % was calculated.

Statistical analyses were performed for both the primary outcome O3I and the secondary outcomes (anthropometric data, dietary intake and other fatty acids in erythrocytes). Data are presented as mean values and standard deviations. Distribution of all data was assessed using the Shapiro–Wilk test and Gaussian distribution. Based on the distribution of data, differences in baseline characteristics were assessed using the Mann–Whitney *U* test or unpaired *t* test for continuous variables and the *χ*^2^ test for nominal variables. Further analysis was performed with data obtained from all participants that arrived at the first and second day of examination (n_EX_ 6, n_EXCO_ 3). For analysis, not normally distributed data were log transformed, except C18 : 2*n*-6tt which was square root transformed, and differences were assessed using two-factor repeated measures ANOVA using time (*t*_0_ and *t*_12_) intervention (EX and EXCO). If statistically significant differences between groups were detected, a *post hoc* analysis with Bonferroni correction was performed within both groups. *P* values of <0·05 were considered as significant. All statistical analyses were carried out using SPSS software (version 23.0; SPSS Inc.).

## Results

### Baseline characteristics

Data analysed were obtained from a total of seventy-two participants (68 % female, 32 % male). Due to health and personal reasons, the study had a dropout of nine subjects (*n*_EX_ 6, *n*_EXCO_ 3). Baseline characteristics of the study population are shown in Table 2. The mean age was 59·2 (sd 6·2) years, and the average BMI was 27·7 (sd 5·28) kg/m^2^. There were no differences in anthropometric parameters or blood lipids between both groups. The study population was slightly hypercholerosterolaemic.

**Table 2.** Baseline characteristics of all participants* (Mean values and standard deviations)

	Exercise (*n* 36)		Exercise + CO (*n* 36)		Group difference: *P*
Measure	Mean	sd	Mean	sd	
Sex (f/m)	24/12	–	25/11	–	0·80
Age (years)	59·70	6·39	58·5	5·69	0·51
Height (m)	170·9	8·73	171·3	8·74	0·89
Body weight (kg)	81·9	18·6	80·7	20·3	0·61
BMI (kg/m^2^)	28·00	5·51	27·28	5·26	0·61
SBP (mmHg)	129·4	14·7	134·1	20·1	0·27
DBP (mmHg)	76·2	6·16	78·6	7·91	0·15
Pulse (bpm)	74·6	9·58	73·8	8·25	0·71
Total cholesterol (mmol/l)	6·15	1·01	6·26	0·96	0·97
HDL-cholesterol (mmol/l)	1·75	0·40	1·76	0·47	0·76
LDL-cholesterol (mmol/l)	3·89	0·82	3·93	0·76	0·83
TAG (mmol/l)	1·31	0·63	1·23	0·39	0·56

CO, *Calanus finmarchicus* oil; f, female; m, male; SBP, systolic blood pressure; DBP, diastolic blood pressure.

* Distribution of sexes between groups was analysed using the *χ*^2^ test. All other group differences were assessed with the Mann–Whitney *U* test or unpaired *t* test.

### Dietary intake from background diet

The intake of PUFA and especially EPA and DHA showed high variability at both time points (Table 3). Throughout the study, the intake of individual and total fatty acids did not change significantly. Additionally, all other dietary variables did also not change significantly. However, there was a non-significant trend to a lower energetic intake of about 837 kJ (200 kcal) at the end of the intervention in both study groups.

**Table 3.** Dietary energy and nutrient intake calculated from 3-d dietary records at the beginning (0) and at the end (12) of the intervention as well as absolute changes (Δ)* (Mean values and standard deviations)

Parameters		Exercise (*n* 30)		Δ Exercise		Exercise + CO (*n* 33)		Δ Exercise + CO		
	*t*	Mean	sd	Mean	sd	Mean	sd	Mean	sd	Group difference: *P*
Energy intake (kcal/d)†	0	2024	409	–217	455	1924	437	–147	476	0·55
	12	1806	365			1777	415			
Protein (%/d)	0	16·1	3·42	0·86	3·84	15·6	2·99	1·05	3·68	0·84
	12	16·9	3·37			16·7	3·26			
Fat (%/d)	0	36·2	7·47	0·69	7·97	38·1	6·69	–2·51	9·04	0·14
	12	36·9	6·00			35·6	7·48			
Carbohydrates (%/d)	0	42·6	9·45	–2·76	8·36	41·92	6·09	0·47	8·72	0·08
	12	39·8	6·45			42·39	7·86			
Fibre (g/d)	0	23·4	7·79	–4·08	6·79	22·0	6·47	–0·78	8·03	0·15
	12	19·3	6·08			21·2	8·80			
SFA (g/d)	0	35·7	13·6	–4·67	15·7	36·9	15·5	–6·93	15·2	0·69
	12	31·0	11·2			29·9	11·1			
MUFA (g/d)	0	28·9	12·5	–2·99	11·9	27·3	9·61	–3·41	12·01	0·77
	12	26·0	11·2			23·9	9·29			
PUFA (g/d)	0	11·9	3·96	0·21	5·01	11·8	5·64	–1·26	4·07	0·21
	12	12·1	5·75			10·5	5·26			
DHA (g/d)	0	0·29	0·34	0·11	0·46	0·23	0·30	–0·4	0·35	0·41
	12	0·39	0·47			0·19	0·19			
EPA (g/d)	0	0·27	0·54	–0·01	0·37	0·18	0·41	0·09	0·23	0·17
	12	0·30	0·41			0·14	0·19			

CO, *Calanus finmarchicus* oil; *t*, times in weeks.

* *P* values represent time × intervention interaction analysed with two-way repeated-measures ANOVA.

† To convert kcal to kJ, multiply by 4·184.

### Tolerability of the *Calanus finmarchicus* oil capsules

At the beginning of the study, one participant reported suffering from diarrhoea after taking the CO capsules. Despite the instructions to take the capsules with food, this participant took the capsules on an empty stomach. After starting taking the capsules as suggested, the symptoms disappeared. Otherwise, no adverse symptoms after the CO capsule intake were reported.

### Fatty acid content of erythrocytes and omega-3 index

Comparison of baseline values between the two study groups showed no significant differences in fatty acid levels. In both groups, PUFA values declined in the following order ARA >LA > DHA > DPA*n*-3 = C22 : 4*n*-6 > C20 : 3*n*-6 > EPA > DPA*n*-6 > ALA = C20 : 2*n*-6 > C18 : 3*n*-6. Lowest quantities were observed for SDA. O3I was 6·01 (sd 1·26) % in EX and 6·07 (sd 1·29) % in EXCO (Table 4).

**Table 4.** Fatty acid content of erythrocytes (given as percentage of total fatty acids) at the beginning (0) and at the end (12) of the study and absolute changes study (Δ)* (Mean values and standard deviations)

Fatty acid		Exercise (*n* 30)			Δ Exercise		Exercise + CO (*n* 33)			Δ Exercise + CO		Group difference: *P*
	*t*	Mean	sd	*P t*_0_→*t*_12_	Mean	sd	Mean	sd	*P t*_0_→*t*_12_	Mean	sd	
C14 : 0	0	0·37	0·10	–	–0·02	0·09	0·41	0·12	–	–0·02	0·07	0·90
	12	0·35	0·08				0·39	0·10				
C16 : 0	0	21·8	0·89	–	–0·03	0·48	21·5	0·85	–	0·09	0·37	0·23
	12	21·7	1·00				21·6	0·79				
C16 : 1*n*-7t	0	0·11	0·04	–	0·00	0·02	0·14	0·05	–	0·00	0·02	0·68
	12	0·11	0·04				0·14	0·05				
C16 : 1*n*-7	0	0·42	0·21	–	–0·05	0·13	0·41	0·18	–	–0·06	0·07	0·46
	12	0·38	0·18				0·35	0·16				
C18 : 0	0	16·5	0·94	–	0·11	0·60	16·7	0·92	–	0·05	0·49	0·68
	12	16·6	0·86				16·8	0·74				
C18 : 1t	0	0·46	0·09	0·09	–0·02	0·07	0·51	0·10	0·07	0·02	0·06	0·02
	12	0·44	0·08				0·53	0·10				
C18 : 1*n*-9	0	15·5	0·96	–	–0·11	0·55	15·4	1·12	–	–0·14	0·43	0·84
	12	15·4	1·00				15·2	1·11				
C18 : 2*n*-6tt	0	0·01	0·01	–	0·00	0·01	0·01	0·02	–	0·00	0·01	0·88
	12	0·01	0·02				0·01	0·02				
C18 : 2*n*-6ct	0	0·04	0·03	–	0·01	0·02	0·04	0·03	–	0·00	0·01	0·16
	12	0·05	0·04				0·04	0·03				
C18 : 2*n*-6tc	0	0·12	0·02	0·43	0·00	0·03	0·13	0·03	<0·001	0·02	0·02	0·01
	12	0·12	0·02				0·15	0·03				
C18 : 2*n*-6	0	11·3	1·54	–	–0·21	0·89	11·3	1·53	–	–0·52	0·99	0·22
	12	11·1	1·55				10·7	1·21				
C18 : 3*n*-3	0	0·19	0·09	–	–0·01	0·06	0·19	0·09	–	–0·02	0·07	0·63
	12	0·19	0·07				0·17	0·07				
C18 : 3*n*-6	0	0·09	0·05	–	0·00	0·04	0·09	0·03	–	–0·01	0·02	0·22
	12	0·09	0·02				0·08	0·02				
C18 : 4*n*-3	0	0·026	0·011	0·83	0·00	0·01	0·027	0·008	0·008	0·01	0·01	<0·001
	12	0·025	0·011				0·038	0·012				
C20 : 0	0	0·19	0·03	–	0·00	0·02	0·20	0·03	–	–0·01	0·01	0·29
	12	0·19	0·04				0·19	0·03				
C20 : 1*n*-9	0	0·28	0·05	–	0·01	0·04	0·26	0·05	–	0·02	0·03	0·18
	12	0·29	0·06				0·28	0·05				
C20 : 2*n*-6	0	0·23	0·04	–	0·00	0·02	0·22	0·03	–	0·00	0·02	0·15
	12	0·23	0·04				0·22	0·03				
C20 : 3*n*-6	0	1·72	0·36	0·02	–0·07	0·14	1·73	0·30	<0·001	–0·14	0·11	0·01
	12	1·66	0·31				1·59	0·28				
C20 : 4*n*-6	0	15·2	1·21	0·36	0·11	0·63	15·3	1·07	<0·001	–0·46	0·61	0·001
	12	15·3	1·35				14·9	0·94				
C20 : 5*n*-3	0	0·90	0·33	0·96	–0·01	0·18	0·92	0·42	<0·001	0·40	0·26	<0·001
	12	0·89	0·30				1·32	0·37				
C22 : 0	0	0·52	0·08	0·81	0·01	0·04	0·53	0·08	0·002	0·02	0·03	0·02
	12	0·52	0·09				0·51	0·08				
C22 : 4*n*-6	0	2·75	0·51	0·49	0·03	0·19	2·70	0·48	<0·001	–0·25	0·22	<0·001
	12	2·78	0·49				2·46	0·42				
C22 : 5*n*-3	0	2·58	0·38	0·07	0·06	0·20	2·67	0·41	<0·001	0·19	0·18	0·03
	12	2·64	0·32				2·86	0·41				
C22 : 5*n*-6	0	0·62	0·23	0·42	–0·01	0·13	0·63	0·18	<0·001	–0·07	0·06	0·02
	12	0·61	0·23				0·56	0·18				
C22 : 6*n*-3	0	5·12	1·03	0·09	0·14	0·43	5·15	1·03	<0·001	0·89	0·45	<0·001
	12	5·26	1·10				6·04	0·94				
C : 24	0	1·37	0·15	–	0·01	0·12	1·36	0·20	–	–0·02	0·09	0·24
	12	1·38	0·16				1·34	0·17				
C24 : 1*n*-9	0	1·60	0·21	–	0·06	0·16	1·51	0·22	–	0·04	0·10	0·69
	12	1·66	0·27				1·55	0·23				
Omega-3 index	0	6·01	1·26	0·24	0·13	0·51	6·07	1·29	<0·001	1·29	0·66	<0·001
	12	6·15	1·32				7·37	1·10				

CO, *Calanus finmarchicus* oil; *t*, times in weeks.

* *P* values represent time × intervention interaction analysed with two-way repeated-measures ANOVA. In the case of significance, statistical differences within groups were detected with Bonferroni’s *post hoc* test.

During the study, a significant increase in O3I occurred in the EXCO group from 6·07 (sd 1·29) to 7·37 (sd 1·10) % (*P* < 0·001). In line, EPA increased from 0·92 (sd 0·42) to 1·32 (sd 0·37) % (*P* < 0·001) and DHA from 5·15 (sd 1·03) to 6·04 (sd 0·94) (*P* < 0·001). Further, relevant increases occurred in SDA from 0·027 (sd 0·008) to 0·038 (sd 0·012) % (*P* = 0·008) and DPA*n*-3 from 2·67 (sd 0·41) to 2·86 (sd 0·41) % (*P* < 0·001). In contrast, levels of most *n*-6-PUFA slightly decreased in consequence of the intervention: ARA decreased from 15·3 (sd 1·07) to 14·9 (sd 0·94) % (*P* < 0·001), C22 : 4*n*-6 from 2·70 (sd 0·48) to 2·46 (sd 0·42) % (*P* < 0·001) and DPA*n*-6 from 0·63 (sd 0·18) to 0·56 (sd 0·18) % (*P* < 0·001).

The EX group showed no physiologically relevant changes in erythrocyte fatty acid levels throughout the intervention.

## Discussion

Current evidence from preclinical studies indicates promising effects of CO on obesity and obesity-related inflammation as well as on blood glucose control and atherosclerosis^(17–19)^. However, the underlying physiological and molecular mechanisms of action are not yet fully understood. An important prerequisite when examining the physiological effects of marine oils is to ensure that active ingredients enter the body in sufficiently high amounts^(11)^. CO contains the *n*-3 PUFA SDA, EPA and DHA. Hence, the investigation of the effect of *n*-3 PUFA from CO on PUFA blood levels is necessary to understand the mode of action of this novel marine oil.

In evaluating the effect of fatty acids, several factors have to be considered with one of the first being the chemical binding form^(11)^. Commercially available marine *n*-3 PUFA supplements contain fatty acids in the form of TAG, ethyl esters or phospholipids. Contrary to that, >80 % of fatty acids in CO are bound to fatty alcohols, which classifies them as wax esters. Overall wax esters have been discussed as both poorly digestible and bioavailable in mammals^(20)^ and larger amounts are reported to cause gastrointestinal symptoms in humans^(21)^. In the present study, CO was well tolerated. Only one case reported gastrointestinal discomfort (diarrhoea) after ingesting the CO capsules on an empty stomach in the first week of intervention. Those symptoms disappeared immediately after the participant started following the instructions to take the capsules together with a meal. With regard to the bioavailability of wax esters, a single-dose study conducted by Cook *et al.* demonstrated that the bioavailability of wax esters from CO is comparable with ethyl esters from fish oil^(9)^. In the mentioned study, the bioavailability of 260 mg EPA and 156 mg DHA from 4 g of CO *v*. 465 mg EPA and 375 mg DHA from 1 g of ethyl esters from fish oil were compared over a 72 h. Although the dose of EPA + DHA from fish oil was almost twice as high as from CO (840 *v*. 416 mg), no significant differences in bioavailability were found. Moreover, plasma levels of EPA remained even higher in the CO group 24–72 h after intake, indicating that wax esters may be a highly available EPA/DHA source. However, the effect of a longer-term CO intake on EPA and DHA blood levels has not been studied.

In the present study, a 12-week intake of 2·0 g CO, providing about 200 mg of EPA + DHA, led to a significant increase of the O3I from 6·07 (sd 1·29) to 7·37 (sd 1·10) %, which is an increase of 1·29 (sd 0·66) %. In contrast, no difference was found in the control group that performed exercise only, indicating that engagement in a moderate exercise programme does not negatively influence the O3I.

With regard to the low EPA + DHA dose used in this study, there are only four studies using comparable doses^(16,22–24)^. Köhler *et al.*^(16)^ investigated the effect of DHA + EPA from enriched sausages containing 250 mg/d EPA + DHA bound as ethyl esters and 250 mg/d ALA bound as TAG. A control group received sausages containing only 250 mg/d of ALA. After a study period of 8 weeks, a 1·5 % increase in O3I from 4·18 (sd 0·54) to 5·72 (sd 0·66) % was reported for the group consuming the enriched sausages, while no difference was observed for the control group. Another study reported significant improvements of the O3I after supplementing different doses of EPA + DHA using krill oil^(24)^. In krill oil, EPA + DHA are mainly bound in phospholipids. Two hundred and 400 mg/d EPA + DHA, respectively, led to an O3I increase from 3·56 (sd 0·82) to 4·19 (sd 0·79) % and 4·0 (sd 0·88) to 5·17 (sd 0·96) %, respectively^(24)^. Flock *et al.*^(23)^ reported an 1·88 (sd 0·23) % O3I increase (4·29 (sd 0·22) to 6·19 (sd 0·23) %) after a 20-week supplementation of about 300 mg/d TAG-bound EPA + DHA, while Sarter *et al.* reported an increase of 1·7 % (3·9 (sd 1·0) to 4·8 (sd 0·8) %) after 16-week intake of 254 mg/d TAG-bound EPA + DHA^(22)^. Compared with the four previous studies, participants from this study showed an overall better EPA + DHA supply status with baseline values of about 6 %. This is of interest because it has been demonstrated that the response of O3I to *n*-3 PUFA supplementation is dependent on the baseline O3I, with lower O3I leading to greater O3I responses^(25,26)^. Nonetheless, the low dose of EPA + DHA from CO still successfully improved the O3I to an extent close to improvements observed in comparable studies with populations that had substantially lower baseline O3I values. Noteworthy, increases of only 1 % were reported to already decrease the risk for sudden cardiac death^(27)^.

However, the effect of CO supplementation on the O3I is unlikely to be solely due to the gastrointestinal uptake of preformed EPA and DHA. It is more likely that SDA also contributes to the O3I increase as SDA is the most abundant *n*-3 PUFA in CO and one of the precursors in the metabolic pathway of EPA and DHA synthesis. When compared among each other, 2·0 g of CO provided 124 mg SDA but only 109 mg EPA and 87 mg DHA. In a recent study, we investigated the short-term effect of a single-dose intake of 26 g echium oil containing 3 g of SDA and reported significant increases of EPA (47 %) and DHA (21 %) levels in plasma after 72 h^(28)^. Moreover, in a previous long-term study on SDA and O3I, a 16-week supplementation of SDA enriched soyabean oil providing 3·66 g SDA led to a 19·5 % increase of the O3I^(29)^. However, no significant changes in DHA were observed, while EPA and SDA levels increased in erythrocytes. In a recent study, we observed a similar outcome after a 12-week supplementation of 12·9 g ALA^(30)^. In comparison, the 12-week intake of the low *n*-3 PUFA dose in the present study led to increases of 41 % in SDA, 44 % in EPA and 17 % in DHA. Noteworthy, CO also contains a non-negligible amount (about 70 mg/2 g CO) of 22 : 1*n*-11 (cetoleic acid). A recent study demonstrated that cetoleic acid stimulated the conversion of ALA to EPA and DHA in human hepatocytes^(31)^. Therefore, it can be hypothesised that this fatty acid could have contributed to the observed elevated EPA + DHA levels in erythrocytes.

Furthermore, it is important to note that the participants of the present study can be classified as pre-obese according to the mean BMI. This is of importance as obesity is known to affect lipid metabolism^(32,33)^. The slightly elevated cholesterol levels in our study collective also reflect a pre-obese state. Moreover, body weight has been found to be a modulator of the response to supplementation of EPA + DHA where higher body weight is associated with a weaker response to a given amount of EPA + DHA^(23)^. Beyond this background, the present results are of interest as the low dose of EPA + DHA from CO significantly increased the O3I in this pre-obese study collective.

Finally, to fully elucidate the impact of CO supplementation on fatty acid metabolism and EPA + DHA status, further bioavailability studies are needed. Preferably, this should also be combined with a strictly controlled diet and PUFA intake.

### Limitations

As data for this analysis were obtained from a larger interventional trial, there are also some methodical limitations. As mentioned above, when evaluating PUFA uptake, it is preferable to strictly control for dietary intake. That being said, one limitation of our study was the estimation of the EPA and DHA intake via 3-d food logs, as this data are self-reported and prone to over- or underestimation as well as different food selection of the participants. For example, if a participant eats a fish meal on 1 d during the 3-d food logs, this leads to massive fluctuations in the mean EPA + DHA intake levels of the background diet in each group. Moreover, EPA + DHA composition estimated from food logs should be evaluated cautiously as it may be incorrect due to errors in EPA + DHA compositions found in the food databases from nutrient intake calculation software^(34)^. This is also reflected by the variability of the estimated dietary PUFA intake levels at the beginning and at the end of the study. However, as no significant changes in dietary EPA and DHA intake from baseline until after the intervention could be detected among the study groups and, more importantly, no changes in erythrocyte PUFA levels in the EX group were seen, EPA and DHA intake from the background diet can be excluded as a potential confounder in this study. Another limitation of this study is the lack of a placebo group due to the original study design, which encompassed four study groups and did not account for a placebo. Future studies should be conducted with a placebo group.

### Conclusion

This is the first study to show that intake of 2 g CO over a period of 12 weeks significantly improves the O3I in elderly participants engaging in a moderate exercise intervention while exercise alone did not affect the O3I. These data provide the first indication that wax ester-bound *n*-3 PUFA from CO are well absorbed and are suited to cover the *n*-3 PUFA supply. Future studies should investigate the long-term bioavailability of *n*-3 PUFA from CO compared with TAG- or ethyl ester-bound *n*-3 PUFA from fish oil or phospholipid-bound *n*-3 PUFA from krill oil.
